# The Lipid A from the Lipopolysaccharide of the Phototrophic Bacterium *Rhodomicrobium vannielii* ATCC 17100 Revisited

**DOI:** 10.3390/ijms22010258

**Published:** 2020-12-29

**Authors:** Iwona Komaniecka, Katarzyna Susniak, Adam Choma, Holger Heine, Otto Holst

**Affiliations:** 1 Department of Genetics and Microbiology, Institute of Biological Sciences, Maria Curie-Sklodowska University, Akademicka 19, 20-033 Lublin, Poland; kasiasusniak@gmail.com (K.S.); adam.choma@poczta.umcs.lublin.pl (A.C.); 2 Division of Innate Immunity, Research Center Borstel, Leibniz Lung Center, Airway Research Center North (ARCN), German Center for Lung Research (DZL), Parkallee 22, D-23845 Borstel, Germany; hheine@fz-borstel.de; 3 Division of Structural Biochemistry, Research Center Borstel, Leibniz Lung Center, Airway Research Center North (ARCN), German Center for Lung Research (DZL), Parkallee 4a, D-23845 Borstel, Germany; otto.holst@gmail.com

**Keywords:** budding bacteria, galacturonic acid, lipid A, lipopolysaccharide, mannose, structure elucidation

## Abstract

The structure of lipid A from lipopolysaccharide (LPS) of *Rhodomicrobium vannielii* ATCC 17100 (*Rv*) a phototrophic, budding bacterium was re-investigated using high-resolution mass spectrometry, NMR, and chemical degradation protocols. It was found that the (Glc*p*N)-disaccharide lipid A backbone was substituted by a Gal*p*A residue that was connected to C-1 of proximal Glc*p*N. Some of this Gal*p*A residue was β-eliminated by alkaline de-acylation, which indicated the possibility of the presence of another so far unidentified substituent at C-4 in non-stoichiometric amounts. One Man*p* residue substituted C-4′ of distal Glc*p*N. The lipid A backbone was acylated by 16:0(3-OH) at C-2 of proximal Glc*p*N, and by 16:0(3-OH), *i*17:0(3-OH), or 18:0(3-OH) at C-2′ of distal Glc*p*N. Two acyloxy-acyl moieties that were mainly formed by 14:0(3-*O*-14:0) and 16:0(3-*O*-22:1) occupied the distal Glc*p*N of lipid A. Genes that were possibly involved in the modification of *Rv* lipid A were compared with bacterial genes of known function. The biological activity was tested at the model of human mononuclear cells (MNC), showing that *Rv* lipid A alone does not significantly stimulate MNC. At low concentrations of toxic *Escherichia coli* O111:B4 LPS, pre-incubation with *Rv* lipid A resulted in a substantial reduction of activity, but, when higher concentrations of *E. coli* LPS were used, the stimulatory effect was increased.

## 1. Introduction

The budding, purple non-sulphur, Gram-negative bacterium *Rhodomicrobium vannielii* (*Rv*) possesses a unique and complex life cycle, which is characterized by the three different cell types, i.e., budding and swarmer cells, and exospores [1]. Its cell envelope contains lipopolysaccharide (LPS) in its outer membrane, the composition of which has been investigated in several strains [2,3]. In addition, phenol-chloroform-light petroleum (PCP) extraction of the LPS resulted in the isolation of several compounds, namely the LPS and novel hopanoids [4,5], of which 35-aminobacteriohopane-32,33,34-triol that represents the basic moiety.. Two additional hopanoids were substituted at the amino group at C-35 by either tryptophane or ornithine.

Additionally, the structure of the phosphate-free lipid A from *R. vannielii* ATCC 17100 (*Rv*) was investigated [6], however, its structure could not be determined completely. It consisted of a central β-(1′→6)-linked Glc*p*N disaccharide, some 30% of which were substituted by Man*p* in (l→4′)-linkage at the non-reducing Glc*p*N. The reduction experiments with NaBH_4_ indicated that the reducing Glc*p*N might not be substituted at C-1. The mannose-substituted backbone structure of the lipid A was proposed as β-Man*p*-(l→4)-β-Glc*p*N-(1→6)-Glc*p*N. 16:0(3-OH) was linked to the amino group of the reducing Glc*p*N. The residue at the amino group of the non-reducing Glc*p*N could not be identified. Holst and co-workers [6] found *O*-acyl residues, as follows: 14:0(3-*O*-14:0), 14:0(3-OH), 22:1, and *O*-acetyl groups. All of the 3-OH fatty acids had *R*-configuration. The position of double bond in 22:1 was established as Δ^14^. Furthermore, Holst and co-workers [6] indicated that Man*p* was not substituted.

Because this lipid A structure was incomplete and mostly based on chemical analysis and GC-MS data, we decided to re-investigate it, applying modern NMR, mass spectrometry, and chemical degradation protocols that allow for us to report a revised structure now. Additionally, the comparative analysis of genes that are responsible for lipid A backbone biosynthesis was done. The selected *Rv* gene sequences were compared to the analogous sequences that were described for *Rhizobium leguminosarum* bv. Viciae and *Mesorhizobium loti* used as reference strains. In addition, the endotoxic properties of *Rv* lipid A were investigated at the model of human mononuclear cells (MNC). A biologically active, bi-phosphorylated Glc*p*N-based lipid A of *Escherichia coli* O111:B4 LPS was used in these studies.

## 2. Results

**Figure 1:**
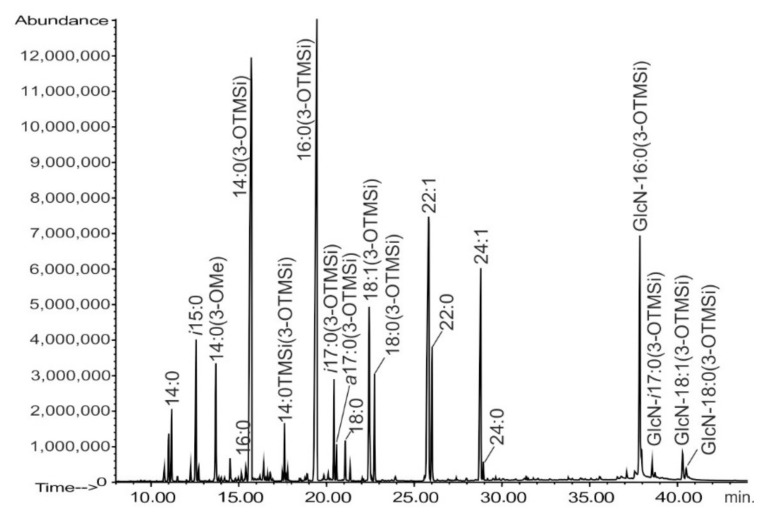
Chromatogram (total ion current) of fatty acids (trimethylsilyl (TMSi) derivatives of methyl esters) from *Rv* lipopolysaccharide (LPS) (2 M HCl/methanol, 85 °C, 18 h; TMSi). At retention time 37.86 min, 38.56 min, 40.30 min, and 40.45 min. *N*-[(3-OH)-acyl]-Glc*p*N derivatives are marked. Unmarked signals were considered to be contaminants.

LPS that was extracted from *Rv* cells utilizing the hot phenol/water method was mainly found (>95%) in the water phase. Fatty acid analysis of LPS revealed the presence of a complex set of 3-hydroxy fatty acids, containing from 14 to 18 carbon atoms. Among them, *iso* and *anteiso* isomers, as well as 18:1, were present (Figure 1, Table 1). The most abundant were 16:0(3-OH) and 14:0(3-OH). Among the non-polar fatty acids, a high amount of two long-chain unsaturated ones were present, namely 22:1ω7 and 24:1ω7, accompanied by their saturated forms. The positions of double bond in unsaturated acyl residues were established analyzing *Rv* LPS fatty acids pyrrolide derivatives [7]. All the hydroxyl fatty acids (with the only exception of 14:0(3-OH)) were found to be amide-bound to the lipid A backbone, as confirmed by GLC-MS analysis (Figure 1, region from 36 to 41 min).

During chromatographic analysis of lipid A fatty acids (Figure 1), we found that the solvolysis conditions that were applied were insufficient to quantitatively liberate fatty acids that were bound via amide linkages. Some of them remained in the form of *N*-acyl-Glc*p*N residues. Their trimethylsilyl derivatives appeared late in chromatographic analysis, and they were identified as *N*-3-hydroxymyristylglucosamine (16:0(3-OH)-Glc*p*N, main peak), and *i*17:0(3-OH)-Glc*p*N, 18:1(3-OH)-Glc*p*N (both are small peaks), and 18:0(3-OH)-Glc*p*N (traces). They were present in a relative ratio of 1.00:0.04:0.04:0.01, respectively, as determined from the total ion current peak areas. The EI-MS fragmentation pattern of the main *N*-acyl-Glc*p*N peak (containing three indicative ions *m/z* at 457, 510, and 543) was the same, as described by Bhat and co-workers in the analysis of *R. leguminosarum* lipid A [8]. A very similar spectrum was also presented by Choma and Komaniecka in the work describing lipid A from *Azospirillum lipoferum* [9].

Lipid A that was obtained from *Rv* LPS by mild acid hydrolysis was subjected to sugar analysis. d-Glc*p*N, d-Man*p*, and d-galactopyranosuronic acid (d-Gal*p*A) were identified as the components of the lipid A backbone.

**Figure 2:**
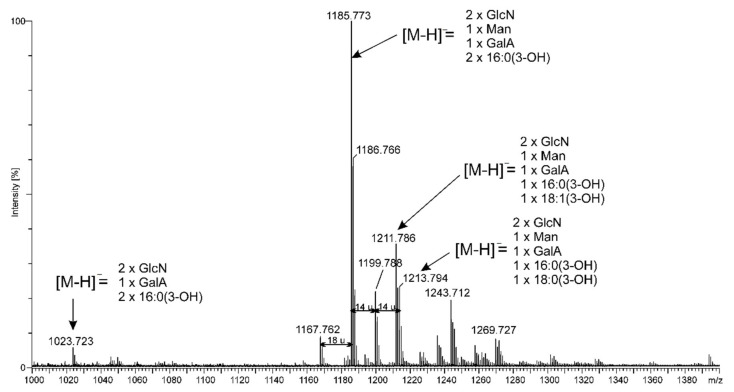
ESI-MS mass spectrum in the negative ion mode of *O*-de-acylated *Rv* lipid A.

**Figure 3:**
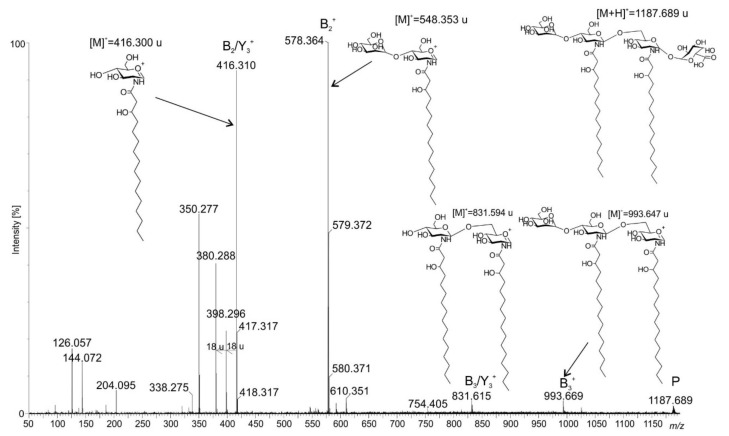
Positive ion ESI-MS-MS spectrum of the signal at *m/z* 1187.689.

**Figure 4:**
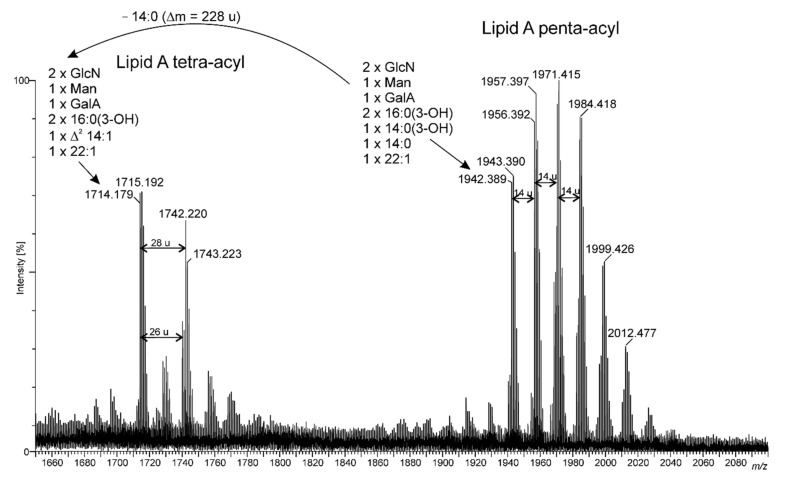
ESI-MS mass spectrum in the negative ion mode of native *Rv* lipid A.

**Figure 5:**
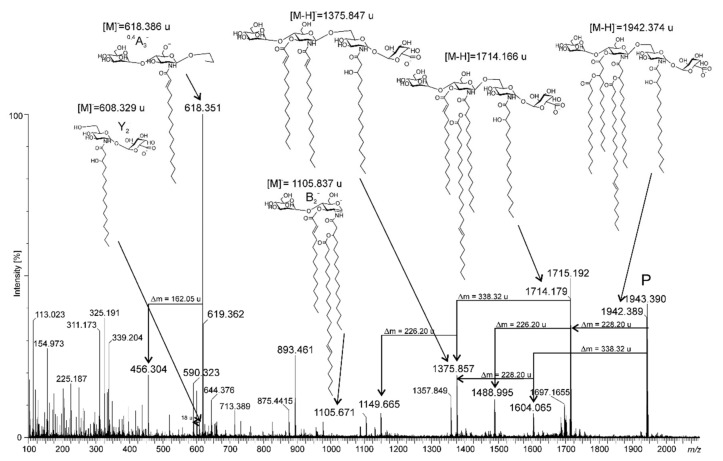
Negative ion ESI-MS-MS spectrum of the signal at *m*/*z* 1942.389.

In order to determine molecular mass and fatty acid distribution in the lipid A from *Rv*, the *O*-de-acylated and native lipid A preparations were analyzed while using electrospray ionization (ESI) mass spectrometry in the negative and positive modes of ionization (Figure 2, Figure 3, Figure 4 and Figure 5). Figure 2 and Figure 4 present the full scan spectra. The spectrum of *O*-de-acylated lipid A (Figure 2) showed intense signals of deprotonated molecules at *m/z* 1185.773, 1199.788, 1211.786, and 1213.794, as well as lower signals at *m/z* 1023.723, 1167.762, 1243.712, and 1269.727. According to our calculations, the main ion at *m/z* 1185.773 corresponded to the lipid A molecules bearing two 16:0(3-OH) amide-linked at the backbone which was built up of a d-Glc*p*N disaccharide, and one residue each of d-Man*p* and d-Gal*p*A (calculated monoisotopic mass [M-H]^−^ = 1185.675 u). The remaining signals (at *m/z* 1199.788, 1211.786, and 1213.794) represented lipid A molecules that contained acyl residues of different length. Only the ion at *m/z* 1023.723 indicated that a few lipid A molecules lacked d-Man*p*. A positive ion was formed as a result of the protonation of *O*-de-acylated lipid A. The ion [M+H]^+^ at *m/z* 1187.689 was fragmented under CID conditions, and the obtained fragmentation pattern allowed for us to determine the positions of d-Man*p* and d-Gal*p*A in the lipid A molecule. Figure 3 shows the deciphered. All of the ions described, particularly type B ions, indicated d-Man*p* to be linked at C-4′. Thus, the d-Glc*p*N disaccharide should be terminated with d-Gal*p*A on the reducing end.

The genomic comparative studies in silico were performed in order to confirm the above observations (Table 2). Proximal part of *Rv* lipid A resembles the mesorhizobial lipid [10,11]. Lipid A of *M. loti* possesses a d-Gal*p*A residue linked at position C-1 of the amino sugar backbone. For this substitution, two enzymes encoded by genes *lpxE* (lipid A 1-phosphatase) and *rgtF* (α-(1,1)-GalA transferase) are responsible. The presence of sequences denoted as: Rvan_2973 (as well as Rvan_3636) having a significant similarity value to the *lpxE*, and Rvan_0660 with a significant similarity to the *rgtF*, clearly indicated that the position C-1 of reducing d-Glc*p*N was substituted by d-Gal*p*A that was linked by α-(1→1)-glycosidic bond. From the other hand, *R. leguminosarum* bv. Viciae 3841 decorates its lipid A backbone with d-Gal*p*A exclusively at its distal part. Two enzymes (LpxF and RgtD) are engaged in this process. Putative ORFs for these genes were not detected in *Rv* ATCC 17100 strain genome (Table 2).

The ESI mass spectrum of native lipid A (Figure 4) exhibited two sets of peaks at *m/z* between 1714 and 1780, and above *m/z* 1942 with prominent signals. The first group of signals corresponded to a tetra-acylated and the second group to a penta-acylated lipid A. At least eight different molecular variants of lipid A could be distinguished in the second (main) group of signals. The difference of 14 u between successive signals proved that diversity originated from the acylation pattern of the lipid A backbone, as well as from primary fatty acids of different chain lengths. This acylation pattern variety mainly originated from ester-linked residues, since the amino groups of both Glc*p*N were almost exclusively substituted by 16:0(3-OH). The mass difference of 228 u between the signals from both groups of ions corresponded to the mass of 14:0 (see Figure 4 and Figure 5), which indicated that signals from *m/z* 1714 to 1780 were due to the degradation (elimination of fatty acid in the ion source) of lipid A molecules carrying five acyl residues or can be considered to be a natural heterogeneity of this lipid A. In summary, the LPS of *Rv* mainly contained penta-acylated lipid A species.

To establish the fatty acid distribution in the native lipid A, we chose the ion at *m/z* 1942.389 to perform ESI MS-MS analysis (Figure 5). The presence of a B_2_¯ ion (at *m/z* 1105.837 in this spectrum (a composition with 16:0(3-*O*-22:1) and 14:1 residues) provided to be the basis for assigning the asymmetric distribution of fatty acids. Similar conclusions could be drawn from the Y_2_¯ ion (at *m/z* 608.328) (see Figure 5, and the inserted chemical formula). Additionally, the presence of ion type ^0,4^A_3_¯ (at *m/z* 618.351) having a characteristic cross-ring fragmentation indicated that position C-3 was not occupied by a 3-OH fatty acid (Figure 5).

Thus, *Rv* native lipid A contained two secondary non-polar fatty acids. One of them, which was connected to the primary fatty acid at C2′-position, belonged to the group of long chain fatty acids and possessed either 22 or 24 carbon atoms. The enzyme transferring this residue from ACP (acyl carrier protein) should be the orthologue of *E. coli* LpxM protein. Computational analysis revealed the presence of Rvan_3546 ORF in the *Rv* genome, which only showed 26% identity and an E value of 4 × 10^−9^ when compared with *E. coli* K-12 *lpxM* (*msbB*). An analogous analysis carried out for *lpxL* (*E. coli* K-12) did not lead to the detection of a similar open reading frame in *Rv*.

NMR spectroscopy of lipid A completed the structural analyses. The signals were assigned by various two-dimensional (2D) techniques, as listed in Materials and Methods, and by comparison to data that were published earlier [12]. The ^1^H NMR spectrum contained nine major signals in the region δ 5.40–4.60, of which those at δ 5.36 and 5.32 originated from the double bonds of 22:1/24:1, and those at δ 5.19 and 5.09 from primary H-3 of 14:0(3-*O*-14:0)/16:0(3-*O*-22:1). The signal at δ 5.12 was assigned to H-3′ of the β-linked Glc*p*N residue of the backbone, C-3′, of which was acylated by 14:0(3-*O*-14:0). The other four signals were assigned to the anomeric protons of α-d-Gal*p*A (residue **C**, δ 5.01), α-d-Glc*p*N (**D**, δ 4.94), α-d-Man*p* (**E**, δ 4.88), and β-d-Glc*p*N (**F**, 4.60). H-2 shifts of residues **D** and **F** were at δ 3.80 and 3.68, respectively. Table 3 summarizes the assignments of all other ring proton and H-6 shifts.

**Figure 6:**
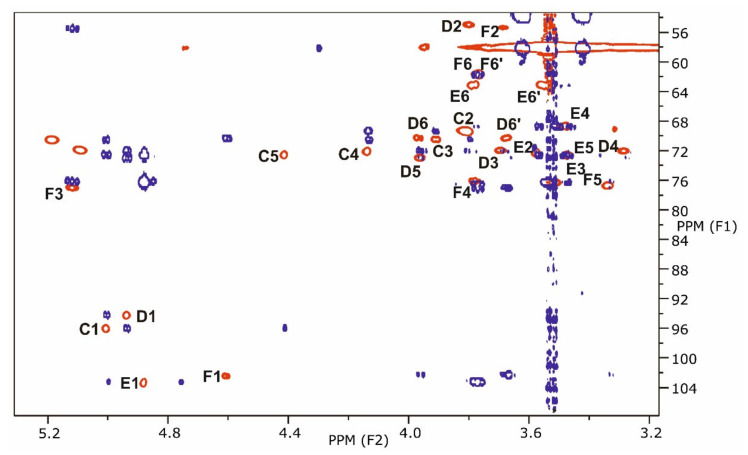
Part of the ^1^H, ^13^C HSQC (red) and ^1^H, ^13^C HMBC (dark blue) spectra of lipid A from *Rv*. Only HSQC signals from backbone sugar residues are marked.

The ^13^C NMR spectrum contained six major signals in the region δ 131.5–94.1, of which those at δ 131.4 and 131.1 originated from the double bonds of 22:1/24:1. The other four signals were assigned to the anomeric protons of α-d-Gal*p*A (**C**, δ 96.0), α-d-Glc*p*N (**D**, δ 94.1), α-d-Man*p* (**E**, δ 103.4) and β-d-Glc*p*N (**F**, 102.2). The signal at δ 76.8 was assigned to C-3′ of the β-linked Glc*p*N residue of the backbone, which was acylated by 14:0(3-*O*-14:0), and those at δ 71.8 and 70.4 to primary C-3 of 14:0(3-*O*-14:0)/16:0(3-*O*-22:1). C-2 signals of residues **D** and **F** were at δ 54.8 and 55.3, respectively. The assignments of all other carbon shifts are also summarized in Table 3 and corresponded cross-peaks of lipid A sugar region were assigned, as depicted in Figure 6.

The data of the ^1^H,^1^H-ROESY spectrum proved the backbone sequence α-d-Man*p*-(1→4)-β-d-Glc*p*N-(1→6)-α-d-Glc*p*N-(1→1)-α-d-Gal*p*A (**E→F→D→C**) by the connectivities H-1 **E**/H-4 **F** (δ 4.88/3.78), H-1 **F**/H-6 **D** (δ 4.60/3.67), H-1 **D**/H-1 **C** (δ 4.94/5.01), and H-1 **C**/H-1 **D** (δ 5.01/4.94). These data were confirmed by HMBC spectroscopy (Figure 6), i.e., H-1 **E**/C-4 **F** (δ 4.88/75.9), H-1 **F**/C-6 **D** (δ 4.60/70.1), H-1 **D**/C-1 **C** (δ 4.94/96.0), and H-1 **C**/C-1 **D** (δ 5.01/94.1).

In a further experiment, lipid A was completely de-acylated by mild hydrazinolysis and hot KOH treatment, and the product was obtained from purification by gel-filtration. Because no other components had been indicated to be present by MS, we expected to obtain the tetrasaccharide α-d-Man*p*-(1→4)-β-d-Glc*p*N-(1→6)-α-d-Glc*p*N-(1→1)-α-d-Gal*p*A. However, a mixture of two compound was yielded, namely this tetrasaccharide and, in somewhat smaller amounts, the tetrasaccharide α-d-Man*p*-(1→4)-β-d-Glc*p*N-(1→6)-α-d-Glc*p*N-(1→1)-β-l-*threo*-hex-4-enuronopyranose (**E’→F’→D’→C’**), clearly proving that, in some amounts of lipid A, the Gal*p*A residue was substituted at C-4, which resulted in β-elimination under the harsh conditions of KOH treatment. Unfortunately, the substituting compound could not be identified, so far.

**Figure 7:**
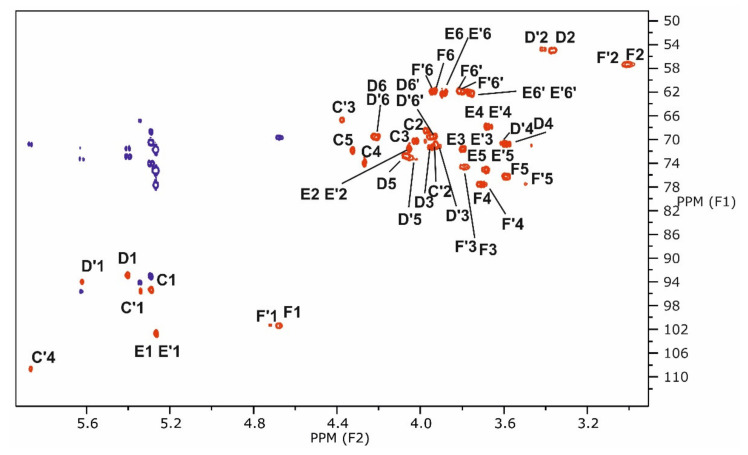
Part of the ^1^H, ^13^C HSQC (red) and ^1^H, ^13^C HMBC (dark blue, only anomeric region) spectra of tetrasaccharides obtained after mild hydrazinolysis and hot KOH treatment of lipid A from LPS of *Rv*. Only HSQC signals from backbone sugar residues are marked.

The structure of this next tetrasaccharide was elucidated by NMR spectroscopy, including an HMBC spectrum that identified the connectivity of H-4 **C’**/C-5 **C’** (δ 5.87/146.1) [12]. Additionally, the HMBC spectrum identified H-1 **C’**/C-1 **D’** (δ 5.34/93.9) (Figure 7). In the ROESY spectrum, the connectivites H-1 **F**(**F’**)/H-6a **D**(**D’**) (δ 4.69(4.72)/4.22(4.22)) and H-1 **E**(**E’**)/H-4 **F** (δ 5.26(5.26)/3.72) were identified. The α-d-Glc*p*N residues (**D**, **D’**) of the tetrasaccharides gave several different ^1^H and ^13^C chemical shifts. Of the β-d-Glc*p*N residues (**F**, **F’**), however, only some ^1^H shift differences could be observed. The chemical shifts of the Man*p* residues remained unchanged. All of the data are summarized in Table 4 and showed in Figure 7.

**Figure 8:**
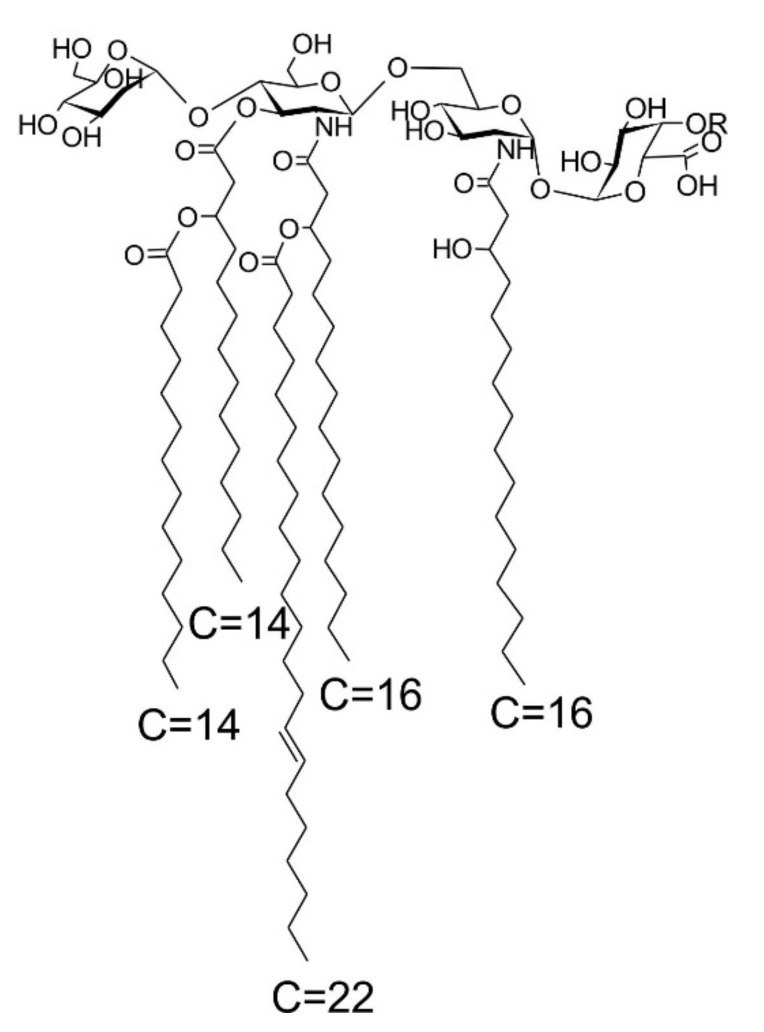
Proposed structure of the main component of *Rv* lipid A (in a molecule corresponding to the ion at *m/z* 1942.388 R = H).

In summary, the structure of the main component of *Rv* lipid A (corresponding to the ion at *m/z* 1942.389 in the ESI-MS spectrum showed in Figure 4) is proposed, as depicted in Figure 8. It should be noted that the NMR data of the de-acylated product indicated the presence of another molecular species containing a, so far, unidentified residue linked to C-4 of Gal*p*A in non-stoichiometric amounts. Four fatty acid residues were linked to the non-reducing β-d-Glc*p*N, and one to the reducing α-d-Glc*p*N (4 + 1 distribution).

**Figure 9:**
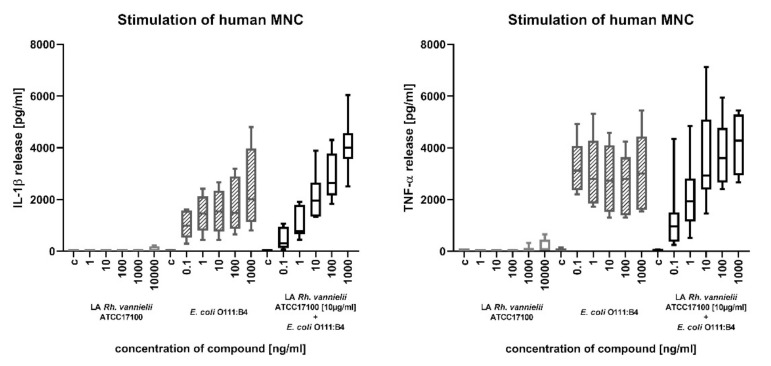
Biological activity of *Rv* lipid A. Human mononuclear cells were either pre-incubated or not with 10 µg/mL *Rv* lipid A (LA *R. vannielii* ATCC 17100) for 1 h and then stimulated with increasing concentrations of *E. coli* O111:B4 LPS or with increasing concentrations of *Rv* lipid A alone. The release of IL-1β (left panel) or TNF-α (right panel) is shown. Error bars represent the standard deviation of the mean (n = 5 independent donors).

The biological activity of *Rv* lipid A was investigated in human mononuclear cells (MNC) (Figure 9). *Rv* lipid A did not possess any significant biological activity in terms of activating human immune cells itself, measured as an ability to induce the production of IL-1β and TNF-α. However, upon pre-incubation of the MNC with 10 µg/mL of *Rv* lipid A, the dose response curve of highly active *E. coli* O111:B4 LPS was shifted. The activity of low concentrations (0.1 and 1 ng/mL of *E. coli* O111:B4 LPS) was decreased, whereas the activity of higher concentrations (100 and 1000 ng/mL of *E. coli* O111:B4 LPS) was increased.

## 3. Discussion

Earlier structural studies on the phosphate-free lipid A of *Rv* [6] showed that this molecule differed considerably from the lipid A structures known then, described e.g., for *Salmonella* and several other Gram-negative bacteria. The authors postulated, that “…the position C-1 of reducing glucosamine can be either not substituted, or the substituent can be removed during the preparation of free lipid A or during reduction with NaBH_4_” [6]. During the current structural studies, utilizing modern analytical techniques and protocols, including high-field two-dimensional NMR spectroscopy and high-resolution MS, it was possible to revise the structure of this lipid A. Particularly, it was found that C-1 was occupied by α-Gal*p*A, and that position C-4′ of β-d-Glc*p*N was substituted by α-d-Man*p*, of which the β-linkage had been proposed in 1983. Additionally, the fatty acid substitution pattern could be identified by high-resolution MS, elucidating a penta-acyl lipid A with a fatty acid distribution of 4 + 1 (Figure 8), which was deprived of a 3-OH-fatty acid at C-3 of the proximal Glc*p*N of the lipid A backbone.

An α-Gal*p*A residue that was linked to C-1 of lipid A has been reported for different bacteria several times [9,10,11]. The transferase encoded by the gene designated as *rgtF* is responsible for the transfer of Gal*p*A from the donor dodecyl-P-GalA to lipid A, according to Brown and co-workers [11]. Similar genes have been identified *in R. leguminosarum*, *M. loti*, *M. opportunistum*, *Caulobacter crescentus*, *Aquifex aeolicus*, *Azospirillum lipoferum* [11], as well as in at least four strains from *Phyllobacterium* [13], and in many bacteria from the class α-proteobacteria, where, in several cases, structural studies have confirmed the presence of α-Gal*p*A at C-1 of lipid A ([14], and this work). Among them are also bradyrhizobia [15], photosynthetic *Bradyrhizobium* BTAi1 [16], and *Rhodopseudomonas palustris* BisA53 [17]. All these strains, together with *Rv*, possess an ortholog of *lpxE* that is required for a dephosphorylation of lipid A precursor at C-1 before the action of RgtF protein (Table 2, and data from NCBI gene database).

Notably, some of the *Rv* lipid A molecules possessed a Gal*p*A that was substituted at C-4 by a, so far, unidentified compound, as clearly shown by the structural analysis of the products that were obtained after mild hydrazinolysis and hot KOH treatment of lipid A. A β-eliminated GalA (β-l-*threo*-hex-4-enuronopyranose) had been identified after such treatment of certain core region and O-antigen structures earlier [12,18]. Contradictory, MS and NMR investigation of lipid A data did not allow for us to identify any additional substituent. However, there were some cross signals in the ^1^H, ^13^C HSQC-DEPT spectrum that could not be assigned.

The presence of Man*p* at C-4′ of *Rv* lipid A was confirmed in this work, as was also observed by Holst and co-workers [6]. However, the β-anomeric configuration proposed in 1983, which was based on the rather preliminary results of chromic oxide treatment, could now be revised to α by the data that were obtained from modern two-dimensional NMR spectroscopy. Man*p* at C-4′ of the lipid A backbone had been identified in several lipid A earlier, also as Man*p* disaccharides or phosphorylated [19,20,21].

In the *Rhodomicrobium* spp. genomes, groups of genes encoding proteins that were associated with the synthesis and modification of lipids A could be detected. Among them, the NCBI database indicates the presence of two sequences encoding acyloxyacyl hydrolases (accession: WP_088342779.1 (206aa) and WP_088346973.1 (159aa)), synonyme “lipid A 3-*O*-acylo deacylase, *pagL*”, the protein that is responsible for removing an acyl substituent from C-3 of the lipid A precursor. These sequences have not an obvious equivalent (counterpart) in the genome of *Rv*. While using BLAST (tblastn), we could conclude that the gene Rvan_3506, annotated in the genome of the strain ATCC 17100 as encoding for ATP/cobalamin adenosyltransferase, possessed a fragment containing motives characteristic for *pagL* (Accession: CP002292.1). However, to demonstrate its modifying effect on *Rv* lipid A, mutagenesis inactivating the above mentioned gene should be carried out, which represents activities that go beyond the subject of the presented work.

The lipid A from *Rv* did not possess any significant stimulatory activity in human mononuclear cells, as seen in Figure 9. Based on the proposed structure, this was not unexpected, since lipid A molecules that differ from the canonical six-fold acylated *E. coli* lipid A structure are usually much less or not active at all [22]. However, the effect on the stimulatory activity of *E. coli* O111:B4 LPS is interesting: at low concentrations of LPS, the pre-incubation with lipid A from *Rv* led to a substantial reduction of activity. This reduction could be explained by the 10–100,000 fold excess of the inactive *Rv* lipid A and the competition of the inactive and active compounds for the molecules that are required for proper LPS signaling (LBP, CD14, TLR4, MD-2) [23]. Surprisingly, when higher concentrations of active LPS (100–1000 ng/mL, leading to a ratio of 1:100 to 1:1000 active vs. inactive molecules) were used, the stimulatory capacity was increased upon pre-incubation with *Rv* lipid A. This effect could hardly be explained by the same competition effect taking place at lower concentrations. Rather, this ratio apparently led to an enhanced accessibility of active LPS molecules, potentially enabling the above-mentioned signaling molecules an easier extraction of LPS molecules from LPS micelles.

## 4. Materials and Methods

### 4.1. Culture Conditions

*Rv* (strain DSM 162, ATCC 17100) was purchased from the DSMZ culture collection, Leibniz-Institute, Braunschweig, Germany. The bacteria were photoheterotrophically grown on *Rhodospirillaceae* medium, containing sodium succinate and ammonium acetate as a carbon and nitrogen source, respectively. The medium was supplemented with cyanocobalamin (Vit. B12), l-cysteine, and resazurin (0.1%, oxonium indicator), and trace element solution containing: ZnSO_4_, MnCl_2_, H_3_BO_4_, CoCl_2_, CuCl_2_, NiCl_2_, and Na_2_MoO_4_. The bacteria were cultivated stationary in 1 L glass bottles containing 900 mL of medium saturated with N_2_, for 8–10 d, in 24 h day-light period (1000 lx) in Fitotron, at 30 °C.

### 4.2. Isolation of LPS

The bacterial cells were harvested, and the cell pellet (125 g wet mass) was washed twice with saline. The bacterial mass was subjected to de-lipidation and enzymatic digestion procedures according to the method that was described by Choma and co-workers (2012) [24]. The LPS was extracted from enzymatically degraded cells while using the hot 45% phenol/water extraction method [25,26], and purified by ultracentrifugation (104,000× *g*, 4 °C, 4 h). The yield of LPS (a water-soluble fraction) was established at the level of 4.4% of dry bacterial mass.

### 4.3. Isolation and Purification of Lipid A

Lipid A was isolated by mild hydrolysis of the water phase deriving LPS (120 mg LPS, 18 mL acetic acid/sodium acetate buffer, pH 4.4, 100 °C, 2.5 h). The hydrolysate was cooled and then converted into a two-phase Bligh-Dyer system, i.e., chloroform/methanol/hydrolysate, 2:2:1.8 (*v*/*v*/*v*), by adding adequate amounts of chloroform and methanol. The chloroform phase containing lipid A was separated by centrifugation and then washed twice with the aqueous phase from a freshly prepared two-phase Bligh–Dyer mixture [21,27]. The lipid A-containing fractions were combined, dried by rotary evaporation, and then stored at −20 °C in chloroform/methanol (3:1, *v*/*v*). As yield, 36 mg (1.2% of the LPS) of pure lipid A was obtained.

### 4.4. Lipid A De-Acylation

For *O*-de-acylation, 1 mg of lipid A was incubated in a mixture containing chloroform/methanol/1 M aqueous NaOH (2:3:1, by vol.) for 1 h at room temperature, according to a previously described method [15,28].

For complete de-acylation, mild hydrazinolysis, followed by 4 M KOH treatment, was applied [29]. Purification involved chloroform extraction and gel-filtration on Sephadex G-10 (0.5 × 40 cm, 10 mM NaHCO_3_).

### 4.5. Fatty Acids and Sugars Analysis

The sugar composition was established after the hydrolysis of lipid A with 2 M trifluoroacetic acid (100 °C, 4 h), after which the liberated monosaccharides were converted into (amino)alditol acetates [30]. The absolute configurations of the monosaccharides were established by the analysis of acetylated *r*-(-)-butyl glycosides according to a procedure of Gerwig and co-workers [31]. The presence of uronic acids in lipid A was established after carboxyl reduction (NaBD_4_, 4 °C, 48 h), hydrolysis, and conversion of the products into alditol acetates.

The qualitative fatty acid composition was established after the methanolysis of LPS with 2 M HCl/MeOH, (85 °C, 18 h). The quantitative fatty acid analysis was performed after hydrolysis of LPS using 4 M aqueous HCl (100 °C, 5 h), the extraction of free fatty acids into chloroform, and methanolysis (0.5 M HCl/methanol, 85 °C, 2 h). In both of the procedures, liberated hydroxy-fatty acid methyl esters were converted into their trimethylsilyl derivatives. To establish the position of double bond in unsaturated fatty acids, the *Rv* LPS fatty acids pyrrolidide derivatives were prepared and analyzed, as was described earlier by Andersson and Holman [7].

Sugar and fatty acid derivatives were analyzed while using a gas chromatograph 7890A (Agilent Technologies, Inc., Wilmington, DE, USA) that was connected to a mass selective detector (MSD 5975C, inert XL EI/CI; Agilent Technologies, Inc., Wilmington, DE, USA) (GLC-MS). Helium was used a carrier gas, with a flow rate of 1.0 mL/min. The chromatograph was equipped with a HP-5MS column (30 m × 0.25 mm). The temperature program was 150 °C/5 min, with 5 °C/min to 310 °C, and held then for 10 min.

### 4.6. Mass Spectrometry

ESI-MS spectrometry was performed with SYNAPT G2-S*i* HDMS instrument (Waters Corporation, Milford, MA, USA) operating in negative and positive ion electrospray mode. The acquisition of the data were performed while using MassLynx software, version 4.1 SCN916 (Waters Corporation, Wilmslow, United Kingdom).

Lipid A samples (native and *O*-de-acylated) were dissolved in chloroform/methanol (3:1, *v*/*v*) at a concentration of 10 µg/µL. For the negative ion mode, a sample of 50 µL was transferred into 2 mL vial and then dissolved in 450 µL of 2-propanol/water/triethylamine (50:50:0.001, by vol.), pH 8.5. For the positive ion mode, 50 µL was dissolved in 450 µL water/acetonitrile/acetic acid (30:10:0.4, by vol.) [21]. The samples were injected by infusion, at a flow rate of 20 µL/min. The capillary and cone voltages were set at 3.0 kV and 40 V for positive electrospray mode and 3.8 kV and 40 V for negative electrospray mode. The source temperature was set to 100 °C, the cone gas was set to a flow rate of 100 L/h, and the desolvation nitrogen gas was used at a flow rate of 600 L/h. For MS/MS experiments, isolated precursor ions were fragmented while using collision voltage of 60 V, 75 V, and 90 V. The data were collected for 120 s for each precursor ion. Mass spectra were assigned with a multi-point external calibration while using sodium iodide (Sigma) in positive and negative ion modes.

### 4.7. NMR Spectroscopy

Homo- and heteronuclear 1D (^1^H, ^13^C) and 2D NMR experiments, i.e., correlation spectroscopy (^1^H,^1^H-COSY), double-quantum filtered phase sensitive correlation spectroscopy (DQF-COSY), total correlation spectroscopy (^1^H,^1^H-TOCSY), rotating frame nuclear Overhauser effect spectroscopy (^1^H,^1^H-ROESY), heteronuclear single quantum coherence-distortionless enhancement by polarization transfer spectroscopy (^1^H,^13^C-HSQC-DEPT), and heteronuclear multiple bond correlation (^1^H,^13^C-HMBC) were recorded on solutions of lipid A in DMSO-^2^H_6_/C^2^H_3_Cl (1:1, *v*:*v*) at 27 °C with a Bruker DRX Avance 700 MHz spectrometer that was equipped with a 5 mm CPQCI multinuclear-inverse cryo probe head with a z gradient and Bruker software. The used frequencies were 700.75 MHz for ^1^H NMR and 176.2 MHz for ^13^C NMR. The NMR spectra of de-acylated lipid A sample, which were obtained from mild hydrazinolysis and hot KOH treatment, were recorded on a solution of ^2^H_2_O. All ^1^H,^13^C spectra were calibrated to internal acetone (δ_H_ 2.225, δ_C_ 31.45).

### 4.8. Assay in Human Mononuclear Cells (MNC)

Peripheral blood MNC from healthy human volunteers (prepared from heparinized blood by gradient centrifugation while using Biocoll, Merck, Darmstadt, Germany) were incubated at a concentration of 1 × 10^6^/mL in 96-well tissue culture plates at a volume of 150 μL using RPMI-1640 medium that was supplemented with 100 U/mL penicillin, 100 μg/mL streptomycin (both PAA Laboratories, GmbH, Cölbe, Germany), and 10% of heat-inactivated FCS (Merck Millipore, Biochrom AG, Berlin, Germany). The cells were then stimulated with either increasing concentrations of lipid A from *Rv* or *E. coli* O111:B4 LPS or pre-incubated with *Rv* lipid A (10 µg/mL) for 1 h and then stimulated with increasing concentrations of *E. coli* O111:B4 LPS. After a culture period of 20 h at 37 °C, the culture supernatants were harvested and the level of IL-1β and TNF-α production was determined while using an ELISA according to the manufacturers’ protocol (Invitrogen GmbH, Karlsruhe, Germany). The data shown represent the mean ± SD from n = 3 independent experiments.

### 4.9. Bioinformatics Tools

Standard BLASTP was used in searching for genes encoding putative proteins that were engaged in the biosynthetic pathway of *Rv* lipid A. *R. leguminosarum* 3841 and *M. loti* MAFF 303099 protein sequences were used as queries in BLASTP searches against *Rv* registered in the Genomes OnLine Database. Individual protein sequences were then compared across their entire span with an on-line Global Alignment tool (using the Needleman-Wunsch algorithm) that was provided by the National Center for Biotechnology Information (NCBI).

## Figures and Tables

**Table 1:** Fatty acid composition of *Rv* LPS. For quantitative analysis, the LPS sample was hydrolyzed while using 4 M HCl. Main components are written in bold face.

Component	Amount [µg/mg LPS]
14:0	3.1
*i*15:0	6.8
16:0	1.0
18:0	2.0
**14:0(3-OH)**	**33.7**
**16:0(3-OH)**	**62.4**
*i*17:0(3-OH)	4.5
*a*17:1(3-OH)	1.5
18:1(3-OH)	4.8
18:0(3-OH)	2.7 ^1^
**22:1ω^7^**	**47.9**
**22:0**	**14.4**
**24:1ω^7^**	**25.1**
24:0	1.0

^1^ Trace amounts of 12:0(3-OH), 13:0(3-OH), *i*15:0(3-OH), were also detected.

**Table 2:** Comparison of genes that are possibly involved in modification of *Rv* ATCC 17100 lipid A with bacterial genes of known functions (reference strains: *R. leguminosarum* bv. Viciae 3841 and *M. loti* MAFF303099).

Gene Name (Putative Protein Function)	*lpxE* (Lipid A 1-Phosphatase)	*lpxF* (Lipid A 4′-Phosphatase)	*rgtD* (4′-Gal*p*A Transferase)	*rgtF* (α-(1→1)-Gal*p*A Transferase)	*rgtE* Bactoprenyl/Phosphate Gal*p*A Transferase
*R. leguminosarum* bv. Viciae 3841 (amino acids number) (reference strain)	RL_RS24225 (RL4708) (244aa) reference gen	RL_RS08140 (RL1570) (257aa) reference gen	RL_RS03600 (RL0684) (473aa) reference gen	ND --------- ---------	RL_RS07630 (RL1470) (338aa) reference gen
*M. loti* MAFF303099 (amino acids number) Expect value	MAFF_RS01140 (271aa) 7 × 10^−41^	ND ------------- -------------	ND ------------ -------------	MAFF_RS01135 (547aa) reference gen	MAFF_RS01125 (244aa) 2 × 10^−33^
*Rv* ATCC 17100 (amino acids number) Expect value (amino acids number) Expect value	Rvan_2973 (223aa) 5 × 10^−7^ Rvan_3636 (234) 6 × 10^−7^	ND ---------- ----------	ND ----------- ------------	Rvan_0660 (537aa) 8 × 10^−69^	Rvan_2632 (263aa) 6 × 10^−25^

**Table 3:** ^1^H and ^13^C chemical shift data of the lipid A backbone tetrasaccharide α-d-Man*p*-(1→4)-β-d-Glc*p*N-(1→6)-α-d-Glc*p*N-(1→1)-α-d-Gal*p*A (**E→F→D→C**). Spectra were recorded on solutions of *Rv* lipid A in DMSO-^2^H_6_/C^2^HCl_3_ (1:1, *v*:*v*) at 27 °C.

Residue	H-1/C-1	H-2/C-2	H-3/C-3	H-4/C-4	H-5/C-5	H-6/C-6	H-6′
**C**, α-d-Gal*p*A	5.01/96.0	3.81/69.2	3.91/70.3	4.14/72.0	4.41/72.5	-/172.5	-
**D**, α-d-Glc*p*N	4.94/94.1	3.80/54.8	3.69/72.0	3.29/71.9	3.96/72.8	3.97/70.1	3.67
**E**, α-d-Man*p*	4.88/103.4	3.57/72.2	3.47/72.5	3.47/68.6	34.7/72.5	3.79/63.1	3.56
**F**, β-d-Glc*p*N	4.60/102.2	3.68/55.3	5.12/76.8	3.78/75.9	3.33/76.6	3.76/61.5	3.76

**Table 4:** ^1^H and ^13^C chemical shift data of the lipid A backbone tetrasaccharides α-d-Man*p*-(1→4)-β-d-Glc*p*N-(1→6)-α-d-Glc*p*N-(1→1)-α-d-Gal*p*A (**E→F→D→C**) and α-d-Man*p*-(1→4)-β-d-Glc*p*N-(1→6)-α-d-Glc*p*N-(1→1)-β-l-*threo*-hex-4-enuronopyranose (**E’→F’→D’→C’**), obtained after mild hydrazinolysis and hot KOH treatment of lipid A from LPS of *Rv*. The spectra were recorded on a solution in ^2^H_2_O at 27 °C.

Residue	H-1/C-1	H-2/C-2	H-3/C-3	H-4/C-4	H-5/C-5	H-6/C-6	H-6′
**D’**, α-d-Glc*p*N	5.62/93.9	3.42/54.5	3.92/70.7	3.61/70.5	4.06/73.1	4.22/69.3	3.95
**D**, α-d-Glc*p*N	5.40/92.8	3.38/54.8	3.95/71.2	3.59/70.5	4.08/72.7	4.22/69.3	3.95
**C’**, hex-4-en-uronopyranose	5.34/95.3	3.93/70.7	4.34/66.7	5.87/108.6	-/146.1	-/170.2	-
**C**, α-d-Gal*p*A	5.29/95.1	3.97/68.4	4.03/70.1	4.27/73.7	4.330/71.8	-/176.4	-
**E, E’** α-d-Man*p*	5.26/102.6	4.05/71.4	3.80/71.4	3.69/67.9	3.89/71.3	3.89/62.0	3.76
**F’**, β-d-Glc*p*N	4.72/101.3	3.01/57.2	3.79/74.6	3.69/77.4	3.49/77.4	3.95/61.6	3.80
**F**, β-d-Glc*p*N	4.69/101.2	3.04/57.3	3.80/74.6	3.72/77.4	3.59/76.0	3.93/61.6	3.80

## Data Availability

The data presented in this study are available on request from the corresponding author.

## Abbreviations

ACP	acyl carrier protein
EI	electron impact ionization
ESI-MS	electrospray ionization—mass spectrometry
Gal*p*A	galacturonic acid
MNC	human mononuclear cells
NMR	nuclear magnetic resonance
*Rv*	*Rhodomicrobium vannielii* ATCC 17100
